# Exosome-mediated delivery of 3-n-butylphthalide rescues microglial energy crisis and ameliorates neuroinflammation in ischemic stroke^☆^

**DOI:** 10.1016/j.neurot.2026.e00977

**Published:** 2026-07-29

**Authors:** Bangyue Wang, Muchen Xi, Keyi Lin, Qinglong Guo, Yuyang Wang

**Affiliations:** a Department of Neurosurgery, Second Affiliated Hospital of Anhui Medical University, Anhui Medical University, Hefei, People's Republic of China; b Department of Neurosurgery, First Affiliated Hospital of Anhui Medical University, Anhui Medical University, Hefei, People's Republic of China; c Anhui Province Key Laboratory of Cognition and Neuropsychiatric Disorders, The First Affiliated Hospital of Anhui Medical University, Anhui, People's Republic of China

**Keywords:** Cerebral ischemia, Mitophagy, Microglia, Energy metabolism, 3-n-butylphthalide

## Abstract

Stroke remains the second leading cause of death and the primary cause of long-term disability worldwide, with ischemic stroke accounting for the majority of cases. Ischemia triggers robust microglial activation, yet the precise regulatory mechanisms underlying microglial functional reprogramming remain incompletely understood. Here, we demonstrate that excessive mitophagy drives metabolic energy failure in microglia following cerebral ischemia, resulting in impaired phagocytosis and exacerbated neuroinflammation. Analysis of single-cell RNA-sequencing data from mouse brains in the sham, transient middle cerebral artery occlusion (tMCAO, mMCAO), and permanent middle cerebral artery occlusion (pMCAO, sMCAO) groups revealed that mitophagy was markedly activated in microglia under sustained ischemia and was associated with impaired phagocytic and cytoskeletal pathways. In vitro oxygen-glucose deprivation (OGD) assays showed that phagocytosis of apoptotic neurons by microglia induced upregulation of Drp1, triggering excessive mitochondrial fission and mitophagy, which caused ATP depletion and reduced clearance capacity. The mitophagy inhibitor 3-methyladenine alleviated inflammatory responses but failed to restore mitochondrial quality. In contrast, 3-n-butylphthalide (NBP) stabilized mitochondrial membrane potential, restored ATP production, and improved microglial phagocytic defects and inflammation. To achieve targeted delivery, we constructed BV2 microglia-derived exosomes encapsulating NBP (BV2^exo^@ NBP), which efficiently enhanced drug accumulation in ischemic lesions and significantly improved neurological outcomes in stroked mice. These results identify excessive mitophagy as a core mechanism underlying microglial energy crisis after cerebral ischemia and provide a mitochondria-targeted therapeutic strategy for ischemic stroke. Importantly, the neuroprotective efficacy, mitochondrial restoration, and anti-inflammatory effects of BM@NEB were fully recapitulated in 18-month-old aged mice, a clinically relevant model that more closely reflects the stroke patient population, supporting the translational potential of this exosome-based therapeutic strategy.

## Graphical abstract

**Figure:**
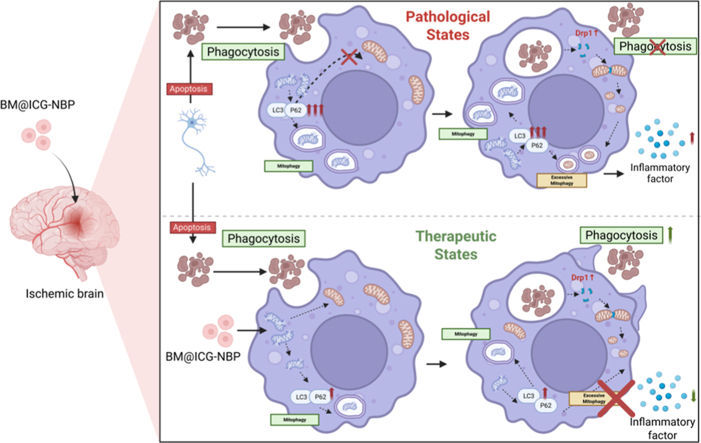

## Introduction

Stroke is the second leading cause of death and the leading cause of adult disability worldwide, among which ischemic stroke caused by arterial occlusion represents the predominant subtype [1]. Insufficient tissue perfusion after cerebral ischemia triggers a cascade of pathological events, including energy metabolic disturbance, excitotoxicity, oxidative stress, inflammatory responses, and apoptosis, ultimately leading to neuronal death and neurological deficits [2]. In this process, microglia, the resident innate immune cells of the central nervous system, are rapidly activated, and their functional states play a decisive role in injury resolution and prognosis [3]. Activated microglia can polarize toward the classic pro-inflammatory M1 phenotype or the alternative anti-inflammatory M2 phenotype, participating in neuroinflammation by releasing pro- or anti-inflammatory mediators, respectively [[4], [5], [6]]. Meanwhile, the phagocytic function of microglia facilitates timely clearance of cellular debris, creating a favorable microenvironment for tissue repair [7].

Mitophagy is a critical cytoprotective mechanism that selectively eliminates damaged mitochondria during cerebral ischemia [8]. However, under extreme conditions, excessive removal of mitochondria can also promote cell death [9]. Microglial functions are highly dependent on flexible energy metabolism. After cerebral ischemia, microglia undergo significant metabolic reprogramming characterized by enhanced glycolysis and weakened oxidative phosphorylation, which is considered a core intrinsic mechanism driving phenotypic and functional switching [2]. Maintenance of energy homeostasis relies on precise regulation of mitochondrial quality, and mitophagy, as a key quality control process, removes damaged mitochondria and sustains cellular energy supply [10]. Under ischemic stress, mitochondrial dysfunction activates mitophagy, which, to a certain extent, helps eliminate impaired organelles, alleviate oxidative stress, and exert protective effects [11]. Nevertheless, emerging evidence suggests that mitophagy acts as a “double-edged sword”: moderate activation is protective, whereas excessive activation may lead to aberrant elimination of functional mitochondria and subsequent cellular energy crisis [12]. For instance, excessive mitophagy contributes to neuronal death and accelerates Huntington's disease progression [13], whereas inhibition of mitophagy exerts renoprotective effects in cisplatin-induced kidney injury [14].

Currently, the role of mitophagy in cerebral ischemia remains controversial. Therefore, elucidating the specific role and regulatory mechanisms of mitophagy during ischemic progression is of great importance. Using single-cell transcriptomic analysis of ischemic mouse brains, we identified microglia as the major cell type undergoing mitophagy and confirmed that excessive mitophagy in microglia leads to phagocytic dysfunction and neuroinflammation. Inhibition of mitophagy or improvement of mitochondrial metabolism effectively alleviates phagocytic defects and inflammatory responses. Based on this mechanism, we further developed a novel therapeutic strategy: a drug delivery system using BV2 cell-derived ^exo^somes encapsulating 3-n-butylphthalide (NBP), which efficiently accumulates in ischemic lesions and improves functional outcomes in stroked mice.

## Materials and Methods

### Animals and ethical approval

Adult male C57BL/6 mice (8–10 weeks old, weighing 22–25 g) were purchased from the Animal Center of Anhui Medical University. Mice were housed in a specific pathogen-free (SPF) environment under a 12-h light/dark cycle at 22 ± 2 °C with free access to food and water. All animal experiments were approved by the Institutional Animal Care and Use Committee (IACUC) of First Affiliated Hospital of Anhui Medical University (approval number: IACU-2502120) and performed in strict accordance with the NIH Guide for the Care and Use of Laboratory Animals. Mice were randomly assigned to groups using a random number table, and all surgical procedures were conducted under sterile conditions.

For aged mouse experiments, male C57BL/6 mice (18 months old, 28–35 g) were purchased from the Animal Center of Anhui Medical University and housed under identical SPF conditions as described above. In vivo and ex vivo were performed as described above for young mice.

### Establishment of cerebral ischemia models

Transient and permanent middle cerebral artery occlusion (tMCAO/sMCAO) was performed using the intraluminal filament technique with minor modifications. In brief, mice were anesthetized with isoflurane (induction: 3%, maintenance: 1.5%). A silicon-coated nylon monofilament (diameter 0.22–0.23 mm, coating length 3–4 mm) was inserted from the external carotid artery into the internal carotid artery to occlude the origin of the middle cerebral artery.

tMCAO (mMCAO): reperfusion was achieved by withdrawing the filament after 60 min of occlusion.

sMCAO: the filament was left in place to establish permanent ischemia.

Sham group: identical surgical procedures were performed without filament insertion.

Cerebral blood flow was monitored by laser Doppler flowmetry to confirm successful occlusion (reduction >80% from baseline). Body temperature was maintained at 37 ± 0.5 °C using a heating pad throughout the surgery. Mice were allowed to recover with free access to food and water.

### Cell culture and oxygen-glucose deprivation (OGD) model

#### Primary microglia culture and OGD treatment

Primary microglia were cultured in Dulbecco's Modified Eagle's Medium (DMEM) supplemented with 10% fetal bovine serum and 1% penicillin-streptomycin at 37 °C for 14 days. Microglia were purified by shaking and used for subsequent experiments.

For in vitro ischemia modeling, OGD was performed as follows: culture medium was replaced with glucose-free DMEM, and cells were incubated in a hypoxic chamber (94% N_2_, 5% CO_2_, 1% O_2_) for 2–4 h.

#### Co-culture model of microglia and apoptotic neurons

The BV2 microglial cell line was used for functional validation. The co-culture system was established as follows:

Apoptosis induction: HT22 neuronal cells were exposed to UV-C irradiation (Stratalinker) for 4 h to induce apoptosis.

Co-culture: apoptotic neurons were added to BV2 microglia for subsequent phagocytic and phenotypic analysis.

### Drug treatment and intervention

In vitro intervention:

3-methyladenine (3-MA): cells were pretreated with 5 mM 3-MA (Sigma-Aldrich) for 1 h before OGD [15].

dl-3-n-butylphthalide (NBP): cells were treated with 10 μM NBP for 1 h before OGD [16].

In vivo administration:

Free NBP: NBP was dissolved in corn oil and administered orally at 60 mg/kg at 2 h after ischemia, once daily for 3 consecutive days [17].

BV2exo@ICG-NBP nanoparticles: exosome-mimetic nanodelivery systems were prepared as described in section 4.1. Mice received BV2exo@ICG-NBP via tail vein injection at an equivalent NBP dose of 60 mg/kg or free ICG-NBP at the indicated time points [17].

#### Preparation and characterization of BV2^exo^@ICG-NBP

Exosome isolation and drug loading: exosomes were isolated from BV2 microglial culture supernatant by differential ultracentrifugation (100,000×*g*, 70 min) or commercial exosome isolation kits. NBP and indocyanine green (ICG) were extruded using BV2 exosomes through 50 nm extrusion membrane.

Characterization: the morphology and size distribution of BV2^exo^@ICG-NBP were characterized by transmission electron microscopy (TEM, JEM 1400, JEOL).

In vivo imaging: BV2^exo^@ICG-NBP was injected via the tail vein into MCAO mice. At 1, 6, 12, and 24 h post-injection, mice were anesthetized and transcardially perfused with PBS, and brains were harvested. Ex vivo fluorescence imaging of the ischemic core and penumbra was performed using an in vivo imaging system (IVIS Spectrum, PerkinElmer).

### Bioinformatic analysis of single-cell RNA-seq (scRNA-seq)

Gene expression data (GSE250245) were retrieved from the Gene Expression Omnibus (GEO) public database. Raw sequencing data were processed using the Cell Ranger pipeline (10x Genomics) and aligned to the mouse reference genome (mm10). Quality control criteria included: (1) removal of cells with <200 or >6000 detected genes; (2) removal of cells with >10% mitochondrial gene expression; (3) exclusion of potential doublets using DoubletFinder. After normalization and scaling, principal component analysis (PCA) was performed, and the top 30 principal components were used for downstream analysis. Unsupervised graph-based clustering was conducted using the Seurat R package (v4.0) in R with a resolution of 0.6, yielding 30 distinct clusters. Cell types were annotated based on canonical marker genes: astrocytes (Gfap, Aqp4), endothelial cells (Pecam1, Cldn5), ependymal cells (Foxj1), excitatory neurons (Slc17a7, Neurod6), inhibitory neurons (Gad1, Gad2), microglia (Cx3cr1, P2ry12), and oligodendrocytes (Mbp, Plp1). Pathway activity scores for mitophagy, TNF-α/NF-κB signaling, interferon-γ response, and inflammatory response were calculated using the AUCell algorithm (AUCell R package). Gene Ontology (GO) biological process and KEGG pathway enrichment analyses were performed using the clusterProfiler package. Pseudotime trajectory analysis was conducted using Monocle 2/3 to reconstruct the temporal dynamics of microglial activation from sham to sMCAO conditions.

### Western blot analysis

Cells or ischemic brain hemispheres were lysed in RIPA buffer supplemented with protease and phosphatase inhibitors. Protein concentration was determined using the BCA assay. Equal amounts of protein (30–50 μg) were separated by 10%–12% SDS-PAGE and transferred to PVDF membranes. Membranes were blocked with 5% non-fat milk for 1 h at room temperature and incubated overnight at 4 °C with primary antibodies: NLRP3 (Cell Signaling, #15101, 1:1000), Drp1 (Cell Signaling, #8570, 1:1000), LC3-II (Cell Signaling, #4108, 1:1000), p62/SQSTM1 (Cell Signaling, #5114, 1:1000), COXIV (Affinity, #AF5468, 1:1000), TOM20 (Affinity, #AF5206, 1:1000), and β-actin (Servicebio, #AC220730003, 1:5000). After washing, membranes were incubated with HRP-conjugated secondary antibodies (Servicebio, #GB23303, 1:5000) for 1 h at room temperature. Protein bands were visualized using an enhanced chemiluminescence (ECL) kit and quantified using ImageJ software.

### Enzyme-linked immunosorbent assay (ELISA)

Commercial ELISA kits were used to measure the levels of IL-6 (#KE10007, Proteintech), TNF-α (#KE10002, Proteintech), and IL-17 (#KE100020, Proteintech) in cell culture supernatants or brain homogenates according to the manufacturers’ instructions. Absorbance at 450 nm was measured using a microplate reader (Bio-Rad). Cytokine concentrations were calculated based on standard curves and normalized to total protein content or cell number.

### Immunofluorescence staining

Cells grown on coverslips were fixed with 4% paraformaldehyde for 15 min, permeabilized with 0.3% Triton X-100 for 10 min, and blocked with 5% bovine serum albumin (BSA) for 1 h. Cells were incubated overnight at 4 °C with primary antibody against Iba1 (microglial activation marker), followed by Alexa Fluor-conjugated secondary antibodies (1:500) for 1 h at room temperature. Nuclei were counterstained with DAPI (1 μg/mL). Images were acquired using confocal laser scanning microscopes (Leica TCS SP8, Zeiss LSM 888).

Mitophagy assay: mitochondria were labeled with MitoTracker Green (100 nM, Invitrogen; Beyotime, #C10600) and lysosomes with LysoTracker Red (50 nM, Invitrogen; Beyotime, #C1046). After incubation at 37 °C for 30 min, cells were either fixed or imaged live. Co-localization of mitochondrial and lysosomal signals was used as an indicator of mitophagy.

Mitochondrial membrane potential (ΔΨm): was evaluated using a JC-1 mitochondrial membrane potential assay kit (Merck, #CS0390-1 KT). In healthy mitochondria, JC-1 forms J-aggregates emitting red fluorescence; in depolarized mitochondria, JC-1 remains in monomeric form emitting green fluorescence. The ratio of red-to-green fluorescence intensity reflects mitochondrial membrane potential integrity.

### Measurement of ATP and ROS levels

ATP content: cellular or tissue ATP levels were detected using an ATP bioluminescence assay kit (Beyotime, #S0026). Samples were lysed and centrifuged, and supernatants were mixed with luciferase reagent. Luminescence intensity was measured using a multimode microplate reader (PerkinElmer). ATP concentration was calculated based on a standard curve and normalized to protein concentration.

Reactive oxygen species (ROS): intracellular ROS levels were detected using the fluorescent probe DCFH-DA. Cells were incubated with 10 μM DCFH-DA at 37 °C for 30 min, washed with PBS, and fluorescence intensity was measured using a fluorometer or flow cytometer (excitation 488 nm, emission 525 nm).

### TTC staining for cerebral infarct volume

Mice were deeply anesthetized and transcardially perfused with PBS. Brains were rapidly removed, frozen at −20 °C for 10 min, and cut into 2-mm coronal sections using a brain matrix. Sections were incubated in 2% 2,3,5-triphenyltetrazolium chloride (TTC) solution at 37 °C in the dark for 15–20 min and fixed overnight in 4% paraformaldehyde. Stained sections were photographed.

### Statistical analysis

All data are presented as mean ± standard deviation (SD) or median (interquartile range) as appropriate. Statistical analyses were performed using GraphPad Prism 9.0 or R 4.2. Normality was assessed using the Shapiro-Wilk test. Comparisons between two groups were performed using Student's t-test (parametric) or Mann-Whitney *U* test (non-parametric). For multiple groups, normally distributed data were analyzed using one- or two-way ANOVA followed by Tukey's or Sidak's post hoc test; non-parametric data were analyzed using the Kruskal-Wallis test followed by Dunn's multiple comparisons test (as specified for AUCell scores). A two-sided P < 0.05 was considered statistically significant (∗P < 0.05, ∗∗P < 0.01, ∗∗∗P < 0.001, ∗∗∗∗P < 0.0001).

## Results

**Fig. 1:**
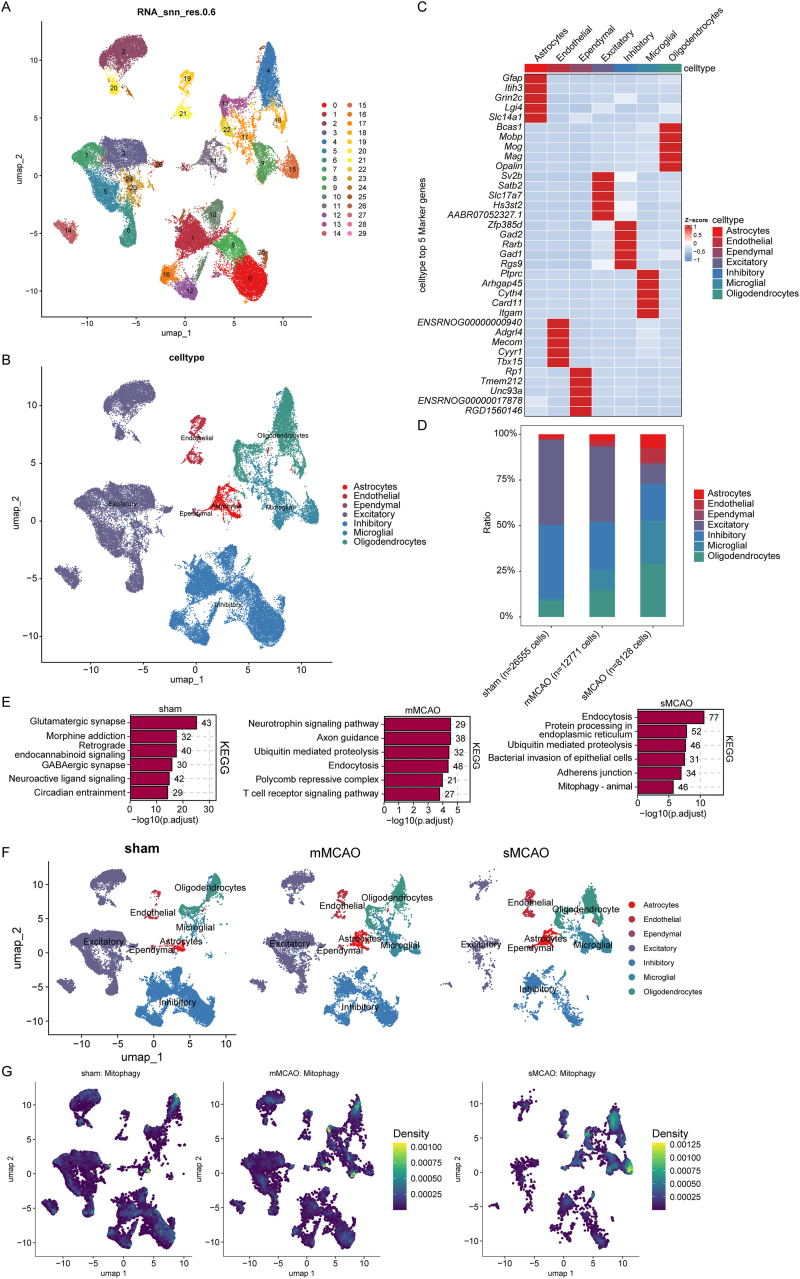
scRNA-seq reveals cellular heterogeneity and mitophagy activation features in the brain after stroke. (A) UMAP dimensionality reduction clustering of all cells (resolution res = 0.6), showing 30 cell clusters. (B) UMAP plot after cell type annotation, classified into 7 major types: Astrocytes, Endothelial, Ependymal, Excitatory, Inhibitory, Microglial, and Oligodendrocytes. (C) Heatmap of top 5 marker genes for each cell type; color scale indicates Z-score normalized expression levels. (D) Stacked bar chart of cell composition proportions across three groups (sham, mMCAO, sMCAO), with total cell counts in parentheses. (E) KEGG pathway enrichment analysis bar chart showing the top 6 significantly enriched pathways in sham, mMCAO, and sMCAO groups; X-axis represents -log10 (adjusted P-value), numbers on the right indicate gene count. (F) UMAP plot grouped by sample origin showing distribution characteristics of sham, mMCAO, and sMCAO cells. (G) Density distribution of mitophagy pathway activity based on AUCell algorithm; color intensity indicates cell density.

To explore cellular heterogeneity, we analyzed public single-cell RNA-seq datasets from sham, mMCAO, and sMCAO mouse brains. After quality control and unsupervised clustering, 30 cell clusters were identified (Fig. 1A) and annotated into seven major cell types: astrocytes, endothelial cells, ependymal cells, excitatory neurons, inhibitory neurons, microglia, and oligodendrocytes (Fig. 1B). Heatmaps of the top five marker genes for each cell type further validated annotation accuracy (Fig. 1C).

Cell proportion analysis revealed significantly increased microglial and astrocyte fractions and decreased neuronal proportions in stroke groups (mMCAO and sMCAO) compared with the sham group, with more pronounced changes in the sMCAO group (Fig. 1D). KEGG enrichment analysis showed that the sham group was enriched in synaptic function-related pathways (e.g., glutamatergic synapse, GABAergic synapse); the mMCAO group was enriched in neuroprotective and axon guidance pathways; whereas the sMCAO group showed significant enrichment in mitophagy, endocytosis, and ubiquitin-mediated proteolysis (Fig. 1E).

Further analysis of mitophagy activity across cell populations revealed significantly elevated mitophagy scores in the sMCAO group compared with sham and mMCAO groups, particularly in microglia and astrocytes (Fig. 1G). These results indicate that cerebral ischemia induces dramatic alterations in brain cellular composition, and sustained ischemia triggers robust activation of mitophagy pathways.

**Fig. 2:**
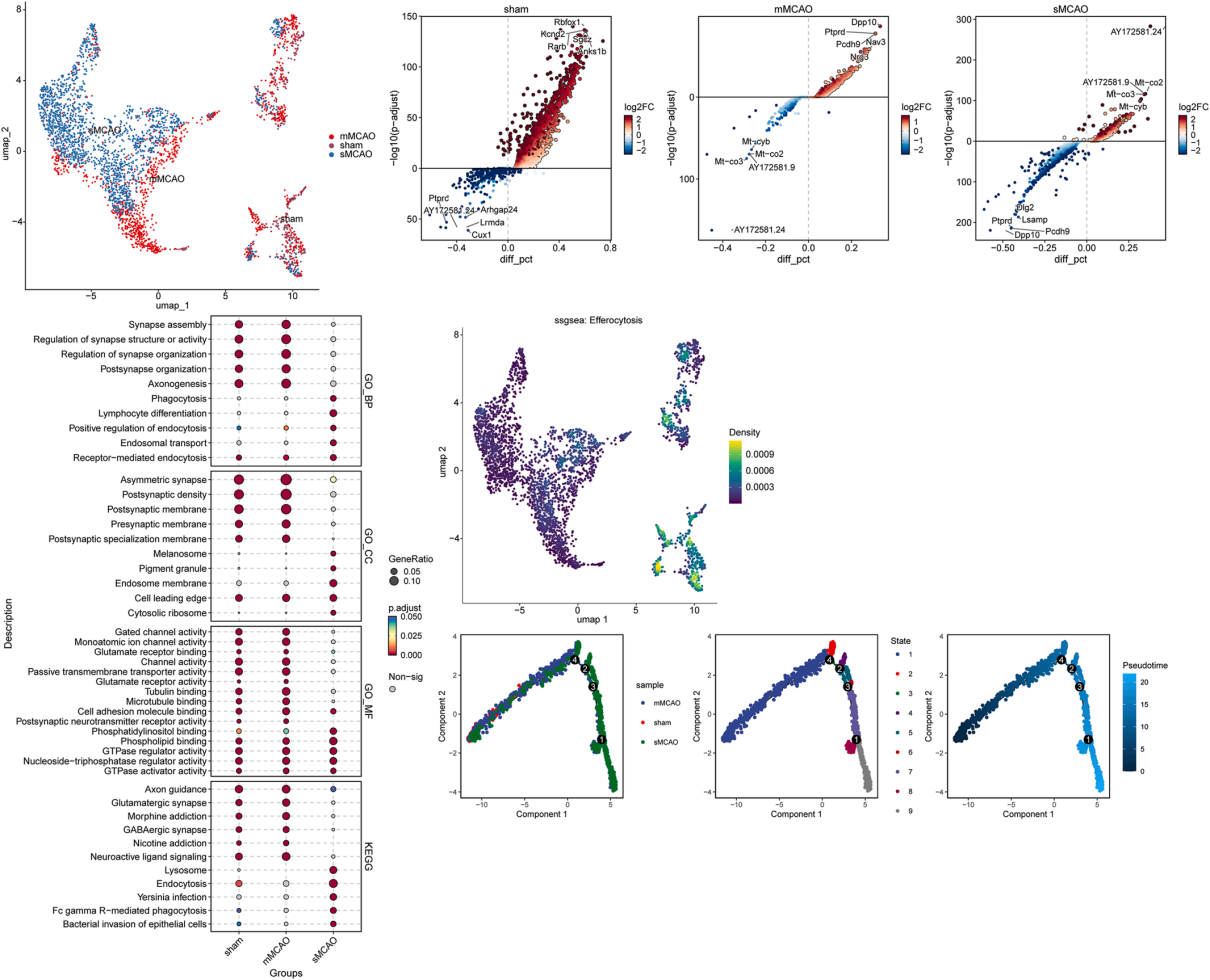
Microglial subpopulation analysis reveals the spatiotemporal association between mitophagy and inflammatory response. (A) UMAP plot of microglial subpopulations; left panel shows all microglia, right panel colored by experimental group (red: mMCAO; blue: sham; cyan: sMCAO). (B) Bubble plot of GO biological process (BP), cellular component (CC), and KEGG pathway enrichment analysis; dot size represents gene ratio, color depth represents -log10 (p-value). (C–F) AUCell score density plots for specific pathways in microglia: (C) Mitophagy; (D) TNFA-SIGNALING-VIA-NFKB; (E) INTERFERON-GAMMA-RESPONSE; (F) INFLAMMATORY-RESPONSE. (G) Violin and box plots of AUCell scores for the four pathways described above, showing score distributions across sham, mMCAO, and sMCAO groups; ∗p < 0.05, ∗∗p < 0.01, ∗∗∗p < 0.001, ∗∗∗∗p < 0.0001 (Kruskal-Wallis test). (H–K) Pseudotime trajectory analysis of microglia: (H) distribution of pseudotime values; (I) distribution of cell states; (J) distribution of sample origin; (K) changes in mitophagy scores along pseudotime.

Given the pivotal role of microglia in stroke, we further performed microglial subset analysis. UMAP visualization showed that sMCAO microglia formed a distinct cluster, whereas mMCAO and sham microglia were more dispersed (Fig. 2A). GO and KEGG analyses revealed that sMCAO microglia were enriched in pathways related to synaptic structure regulation, phagocytosis, lysosomes, and inflammation (Fig. 2B).

AUCell analysis demonstrated significantly enhanced mitophagy, TNF-α signaling, interferon-γ response, and inflammatory response in sMCAO microglia (Fig. 2C–F). Violin plots showed progressive increases in these pathway activities with ischemic severity (sham < mMCAO < sMCAO), with statistically significant differences between groups (Fig. 2G).

Pseudotime analysis reconstructed the transition of microglia from homeostatic (sham) to activated (sMCAO) states (Fig. 2H–J). Mitophagy scores gradually increased along pseudotime and peaked in the sMCAO group (Fig. 2K), suggesting that mitophagy may participate in microglial activation.

**Fig. 3:**
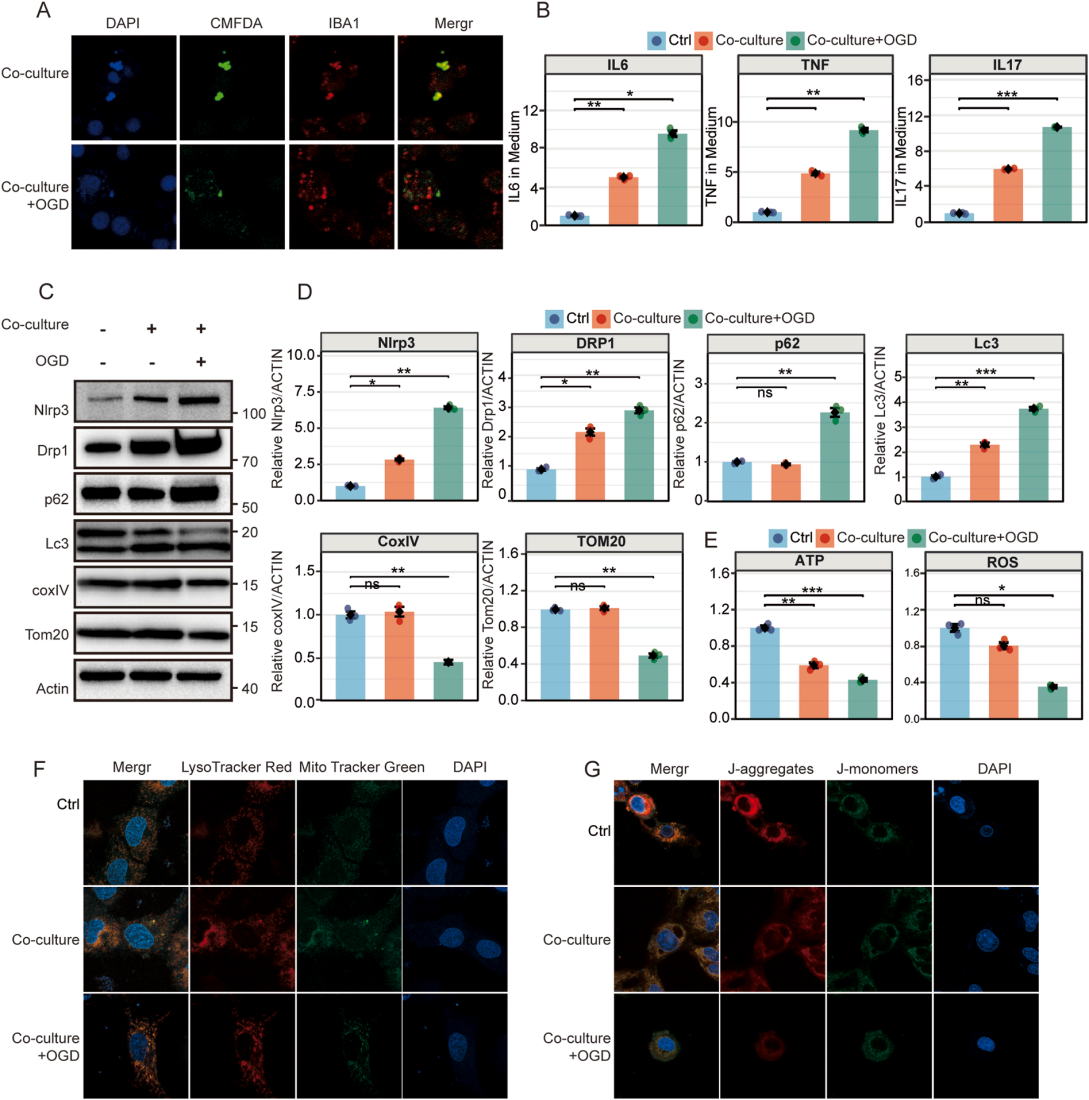
Inflammatory response and mitophagy dysfunction in OGD-induced neuron-microglia co-culture model. (A) Immunofluorescence staining showing microglial activation markers (green) and nuclei (DAPI, blue); red indicates another marker channel. (B) ELISA detection of IL-6, TNF-α, and IL-17 secretion levels in culture medium across groups; ∗p < 0.05, ∗∗p < 0.01, ∗∗∗p < 0.001. (C) Western blot detection of mitophagy-related proteins (Nlrp3, Drp1, p62, LC3) and mitochondrial structural proteins (CoxIV, Tom20). (D) Quantitative statistical bar chart corresponding to panel (C), data expressed as relative ratio (normalized to Actin); ns: not significant, ∗p < 0.05, ∗∗p < 0.01, n = 3. (E) Detection of cellular ATP content and ROS levels across groups; ∗∗p < 0.01, ∗∗∗p < 0.001, n = 3. (F) Immunofluorescence images showing colocalization of lysosomes (LysoTracker Red, red) and mitochondria (MitoTracker Green, green), indicating mitophagosome formation. (G) JC-1 staining for mitochondrial membrane potential; red indicates J-aggregates (healthy mitochondria), green indicates J-monomers (depolarized mitochondria).

To verify single-cell sequencing findings, we established a neuronal-microglial co-culture system subjected to OGD to mimic in vitro ischemia-reperfusion injury. Immunofluorescence showed elevated expression of microglial activation markers after OGD (Fig. 3A). ELISA revealed significantly increased IL-6, TNF-α, and IL-17 levels in the co-culture + OGD group compared with the co-culture alone and control groups (Fig. 3B).

Western blot showed that OGD upregulated mitophagy-related proteins NLRP3, Drp1, and LC3-II, accompanied by p62 accumulation, indicating impaired mitophagic flux (Fig. 3C and D). Decreased expression of mitochondrial structural proteins COXIV and TOM20 indicated severe mitochondrial damage (Fig. 3D). Functional assays showed significantly reduced ATP levels and increased ROS production in the OGD group (Fig. 3E).

Co-staining with LysoTracker Red and MitoTracker Green showed increased co-localization of mitochondria and lysosomes after OGD (Fig. 3F). JC-1 staining revealed significantly reduced mitochondrial membrane potential (J-aggregate/J-monomer ratio) in the OGD group, indicating mitochondrial dysfunction (Fig. 3G).

**Fig. 4:**
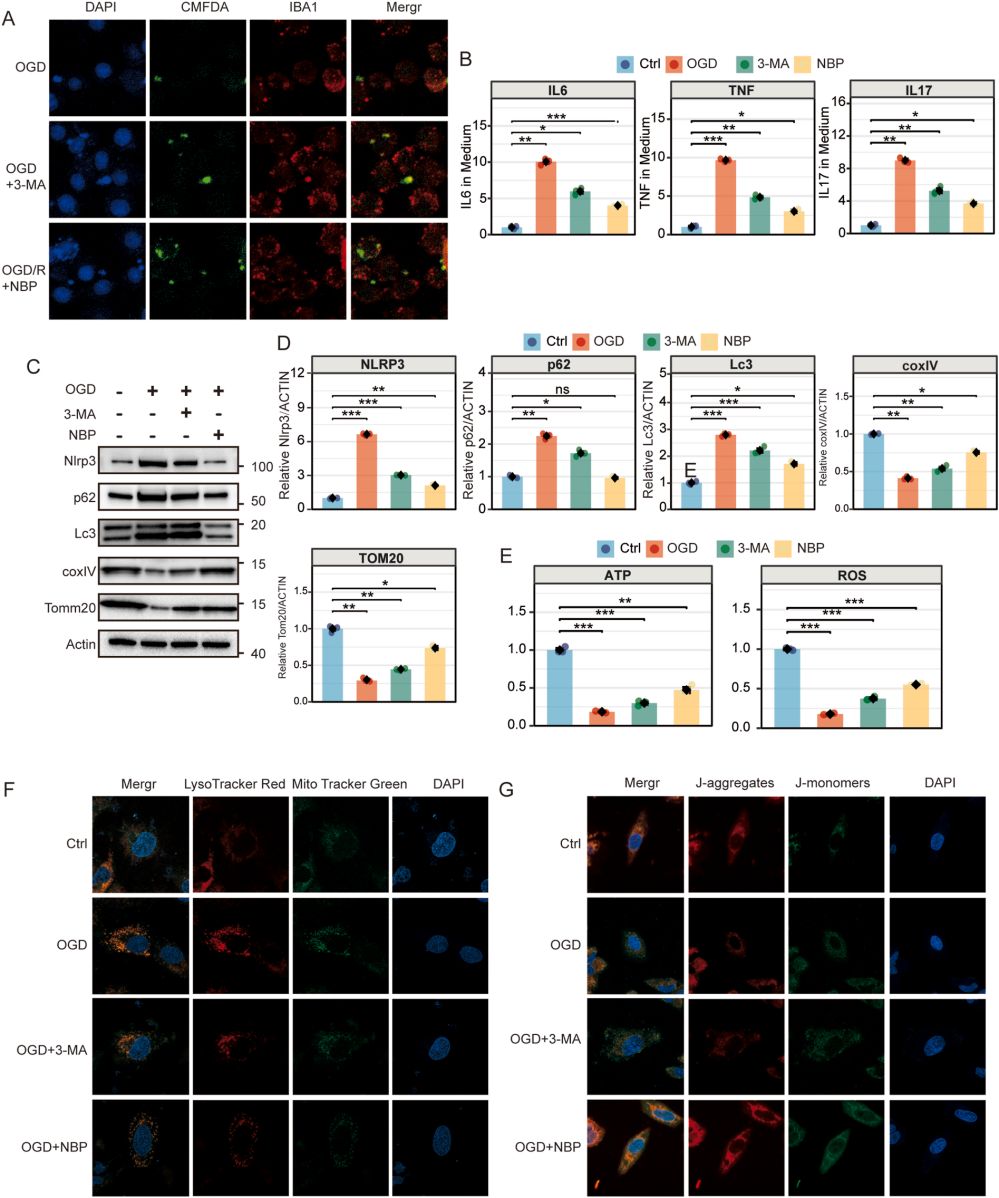
Inhibition of mitophagy or NBP intervention alleviates OGD-induced cellular injury. (A) Immunofluorescence images of different treatment groups (Ctrl, OGD, OGD+3-MA, OGD + NBP). (B) ELISA detection of IL-6, TNF-α, and IL-17 levels across groups, showing inhibitory effects of 3-MA and NBP on cytokine secretion; ∗p < 0.05, ∗∗p < 0.01, ∗∗∗p < 0.001, n = 3. (C) Western blot analysis of NLRP3, p62, LC3-II, CoxIV, and Tom20 protein expression across groups. (D) Quantitative analysis corresponding to panel (C), showing regulatory effects of 3-MA and NBP on mitophagy and mitochondrial structure; ∗p < 0.05, ∗∗p < 0.01, ∗∗∗p < 0.001, n = 3. (E) ATP production and ROS level detection, indicating that interventions improve mitochondrial function; ∗∗p < 0.01, ∗∗∗p < 0.001, n = 3. (F) LysoTracker Red and MitoTracker Green co-staining showing that 3-MA and NBP reduce abnormal mitophagosome accumulation. (G) JC-1 staining showing that 3-MA and NBP treatment restores OGD-induced mitochondrial membrane potential decline.

To investigate the role of mitophagy in OGD-induced injury, we treated cells with the mitophagy inhibitor 3-methyladenine (3-MA) and the positive control drug 3-n-butylphthalide (NBP). Immunofluorescence showed enhanced phagocytic ability in 3-MA and NBP-treated groups compared with the OGD group. ELISA demonstrated that both 3-MA and NBP significantly reduced the secretion of IL-6, TNF-α, and IL-17 compared with the OGD group (Fig. 4A and B).

Western blot analysis showed that 3-MA and NBP effectively inhibited NLRP3 inflammasome activation, reduced LC3-II accumulation, and promoted p62 degradation (Fig. 4C and D). Both treatments partially restored COXIV and TOM20 levels (Fig. 4D), significantly increased ATP production, and decreased ROS levels (Fig. 4E).

Co-localization assays showed that 3-MA and NBP reduced mitochondrial-lysosomal fusion (Fig. 4F). JC-1 staining confirmed that interventions effectively restored mitochondrial membrane potential (Fig. 4G). These results indicate that moderate inhibition of excessive mitophagy or pharmacological intervention alleviates OGD-induced mitochondrial dysfunction and inflammation. Notably, NBP exhibited superior therapeutic efficacy.

**Fig. 5:**
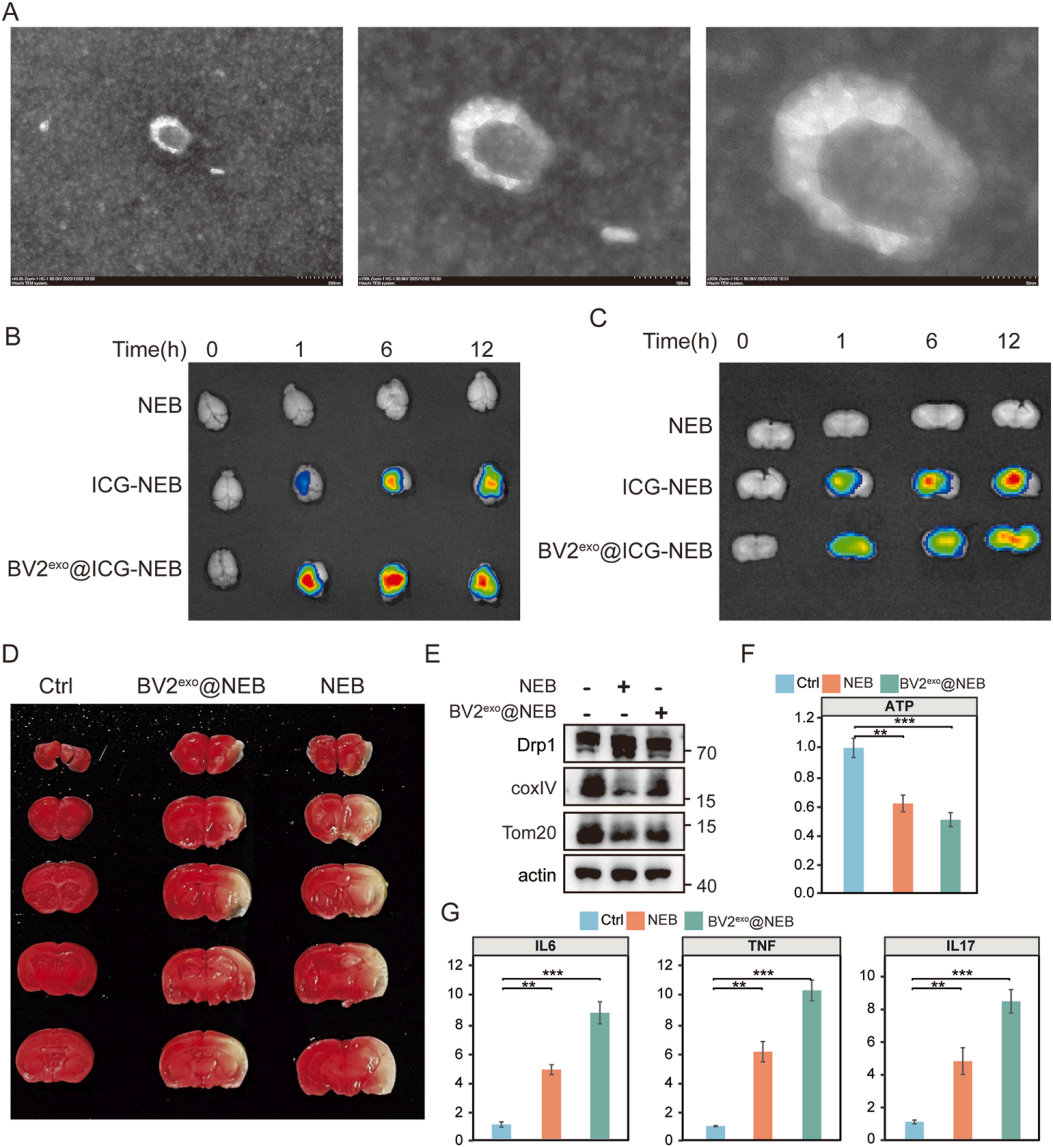
Characterization and therapeutic efficacy of mitochondria-targeted nanomedicine BV2^exo^@ICG-NBP in vivo. (A) Transmission electron microscopy (TEM) images showing the core-shell structure morphology and particle size characteristics of BV2^exo^@ICG-NBP (scale bar: 200 nm). (B, C) Ex vivo brain tissue fluorescence imaging showing distribution characteristics of the drug at different time points (0, 1, 6, 12 h) in (B) the ischemic core region and (C) the ischemic penumbra; heatmap colors (blue→red) indicate fluorescence signal intensity, demonstrating enhanced brain-targeted accumulation and retention capabilities of BV2^exo^@ICG-NBP. (D) Representative TTC staining images; white area indicates infarct tissue, red indicates normal brain tissue, showing differences in cerebral infarct volume among sham control (Ctrl), NBP treatment, and BV2^exo^@NBP treatment groups. (E) Western blot detection of mitochondrial dynamics protein (Drp1) and mitochondrial structural proteins (CoxIV, Tom20) expression levels in brain tissue; numbers on the right indicate relative molecular weight (kDa). (F) Quantitative analysis of brain tissue ATP content, data expressed as relative values; ∗p < 0.05, ∗∗p < 0.01, ∗∗∗p < 0.001, n = 3. (G) ELISA quantitative detection of IL-6, TNF-α, and IL-17 expression levels in brain tissue; ∗∗p < 0.01, ∗∗∗p < 0.001, n = 3.

To achieve precise intervention of mitochondrial dysfunction after cerebral ischemia, we constructed a mitochondria-targeted nanodelivery system, BV2^exo^@ICG-NBP. Transmission electron microscopy (TEM) revealed uniform core-shell nanostructures (Fig. 5A). Ex vivo brain fluorescence imaging showed that BV2^exo^@ICG-NBP, compared with free NBP and ICG-NBP, exhibited strong fluorescent signal enrichment in the ischemic core (Fig. 5B) and penumbra (Fig. 5C) as early as 1 h post-administration, with signal persistence up to 12 h, demonstrating excellent brain-targeting accumulation and prolonged retention.

TTC staining showed that BV2^exo^@ICG-NBP treatment significantly reduced cerebral infarct volume (white areas) after ischemia-reperfusion compared with the MCAO model group, with better neuroprotective efficacy than the free NBP group (Fig. 5D). Western blot confirmed that BV2^exo^@ICG-NBP treatment significantly inhibited expression of the mitochondrial fission protein Drp1 and effectively restored mitochondrial structural proteins COXIV and TOM20, indicating alleviation of post-stroke mitochondrial structural damage (Fig. 5E).

BV2^exo^@ICG-NBP exhibited comprehensive therapeutic advantages in energy metabolism and neuroinflammation: ATP measurement showed that although MCAO markedly reduced brain ATP content, BV2^exo^@ICG-NBP treatment significantly improved energy metabolism, with superior efficacy compared with free NBP (Fig. 5F). Meanwhile, ELISA showed that brain levels of pro-inflammatory cytokines IL-6, TNF-α, and IL-17 in the BV2^exo^@ICG-NBP group were significantly lower than those in the NBP group and closer to sham levels (Fig. 5G). These results demonstrate that BV2^exo^@ICG-NBP, by targeted delivery of NBP to ischemic brain tissue, outperforms free NBP in preserving mitochondrial structure, restoring energy metabolism, and suppressing neuroinflammation, ultimately exerting significant neuroprotection.

**Fig. 6:**
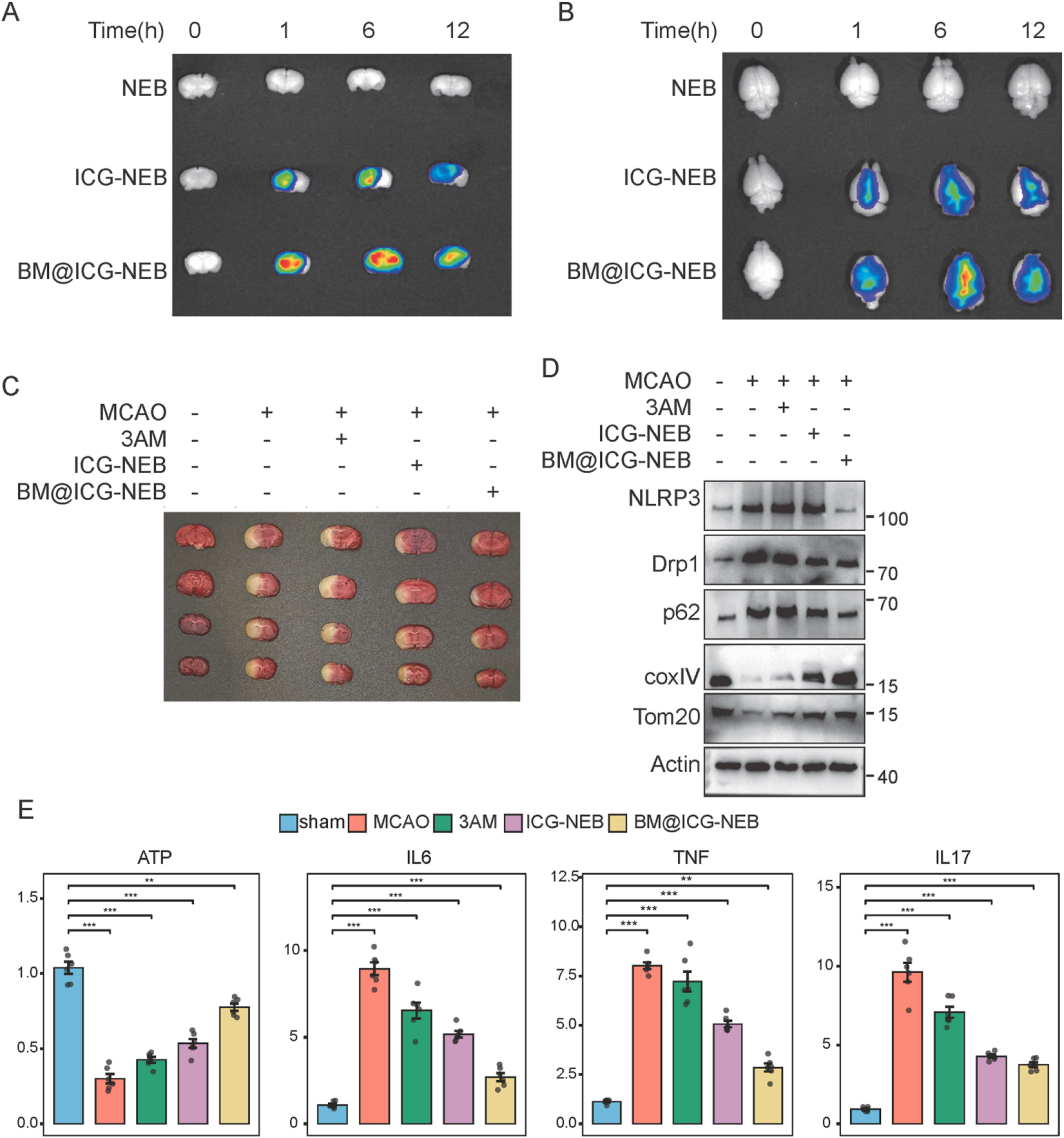
BM@NEB rescues mitochondrial dysfunction and attenuates neuroinflammation in aged mice after ischemic stroke. (A) In vivo near-infrared fluorescence imaging of NEB, ICG-NEB, and BM@ICG-NEB distribution at 0, 1, 6, and 12 h after intravenous administration in aged (18-month-old) mice subjected to tMCAO. Representative images from n = 6 per group are shown.(B) Ex vivo NIR fluorescence imaging of excised brains at 12 h post-administration, confirming selective accumulation of BM@ICG-NEB in the ischemic hemisphere. Representative images from n = 6 per group are shown. (C) Representative TTC-stained brain sections from aged mice in the indicated treatment groups at 24 h post-MCAO. Viable tissue stains red; infarcted tissue appears pale/white. “+” and “-” indicate the presence or absence of MCAO, 3AM, ICG-NEB, or BM@ICG-NEB treatment, respectively. n = 6 per group. (D) Western blot analysis of NLRP3, Drp1, p62, coxIV, Tom20, and Actin (loading control) in peri-infarct cortical tissue from aged mice at 24 h post-MCAO. Quantification is shown in Fig. 6 n = 6 per group.(E) Quantification of ATP levels and pro-inflammatory cytokines (IL-6, TNF-α,IL-17) in brain tissue homogenates from aged mice at 24 h post-MCAO. Data are presented as mean ± SEM, n = 6 per group. ∗∗∗P < 0.001 versus MCAO + Vehicle; ∗∗P < 0.01; ∗P < 0.05 by one-way ANOVA followed by Tukey's post hoc test.

To validate the translational relevance of our findings, we tested BM@NEB in 18-month-old C57BL/6 mice subjected to tMCAO. Aged mice were randomly assigned to five groups: Sham, MCAO + Vehicle, MCAO + 3AM (free NBP), MCAO + ICG-NEB, and MCAO + BM@ICG-NEB. Each MCAO group initially included10 mice; after excluding animals that died within 24 h (mortality: 2–4 per group), n = 6 per group was used for all analyses (Fig. 6).In vivo and ex vivo NIR fluorescence imaging showed that BM@ICG-NEB accumulated in the ischemic hemisphere of aged mice, with peak signal at 6–12 h post-injection, whereas free ICG (NEB) showed minimal brain accumulation (Fig. 6A and B). TTC staining at 24 h revealed that BM@ICG-NEB significantly reduced infarct volume compared with MCAO + Vehicle (P < 0.001) and was superior to free NBP (3AM, P < 0.01; Fig. 6C). ICG-NEB alone had no significant effect.

Western blot analysis of peri-infarct cortex (Fig. 6D) showed that MCAO in aged mice increased Drp1 and NLRP3 expression, and decreased coxIV, Tom20, and p62 levels compared with Sham (all P < 0.001). BM@ICG-NEB reversed these changes: it reduced Drp1 (P < 0.001 vs. MCAO + Vehicle) and NLRP3 (P < 0.001), restored coxIV (P < 0.01) and Tom20 (P < 0.001), and upregulated p62 (P < 0.01). Free NBP showed partial effects, while ICG-NEB had no significant effect, consistent with results in young mice. Functionally, BM@ICG-NEB restored ATP levels (P < 0.001 vs. MCAO + Vehicle) and suppressed IL-6, TNF-α, and IL-17 (all P < 0.001; Fig. 6E). Free NBP modestly increased ATP (P < 0.05) and reduced IL-6 and TNF-α (P < 0.05).Together, these data demonstrate that BM@NEB retains its mitochondrial protective and anti-inflammatory efficacy in aged mice, providing translational validation of this therapeutic strategy.

## Discussion

Mitochondria are the core organelles of aerobic respiration. Hypoglycemic and hypoxic conditions during cerebral ischemia cause mitochondrial dysfunction and structural damage [18,19]. Therefore, agents that target the mitochondrial microenvironment and enhance antioxidant capacity during ischemia represent a promising therapeutic strategy for stroke [20]. Current treatments for ischemic stroke focus mainly on rapid reperfusion to salvage hypoxic tissue, with most studies emphasizing reperfusion injury [21]. However, reperfusion therapies are limited by narrow therapeutic windows and low recanalization rates. Thus, investigating mechanisms underlying ischemic injury holds important complementary value.

The present study focused on cellular injury during the ischemic phase. Single-cell analysis revealed that mitophagy during ischemia occurred predominantly in glial cells, especially microglia. Mitophagy exhibits cell-type-specific regulatory roles in cerebral ischemia: in neurons, PINK1/Parkin-mediated mitophagy during early ischemia removes damaged mitochondria, suppresses oxidative stress, and exerts neuroprotection [22,23]. In vascular endothelial cells within the ischemic core, oxidative stress induces mitochondrial ROS accumulation and tight junction degradation, disrupting blood-brain barrier integrity, whereas enhanced mitophagy alleviates endothelial damage [24]. In microglia, mitophagy bidirectionally regulates inflammation: moderate activation promotes M2 polarization and anti-inflammatory cytokine release, whereas dysfunctional mitophagy drives M1 polarization and excessive IL-1β and TNF-α secretion, exacerbating neuroinflammation [25]. Our study found that severely injured lesions consistently displayed high mitophagy signals. Enrichment analysis revealed that microglia with high mitophagy exhibited impaired cytoskeletal and phagocytic functions, suggesting phagocytic dysfunction.

To further clarify whether mitophagy represents adaptive protection or pathogenic aggravation under ischemic conditions, we established an in vitro microglial phagocytosis model. Results showed that microglial phagocytic capacity decreased under hypoxia, accompanied by elevated inflammation. Investigation of mitophagy revealed that hypomobile microglia exhibited excessive mitophagy. Previous studies reported that sustained apoptotic cell clearance by macrophages induces Drp1 upregulation [26]. We hypothesized that phagocytosis of apoptotic neurons by microglia similarly triggers Drp1 overexpression. Western blot confirmed significantly increased Drp1 expression in microglia phagocytosing apoptotic neurons, which may underlie hyperactivated mitophagy. We also found that excessive mitophagy impaired ATP production, indicating microglial energy failure, which likely accounts for phagocytic dysfunction.

Given that sustained apoptotic cell clearance by macrophages depends on Drp1 [26], we considered direct Drp1 targeting suboptimal. We treated microglia with the mitophagy inhibitor 3-MA and 3-n-butylphthalide (NBP). 3-MA inhibits autophagy by blocking autophagosome formation and is widely used in mitophagy studies [[27], [28], [29]]. NBP is a clinically approved drug for ischemic stroke that improves microcirculation, reduces inflammation, alleviates brain edema and blood-brain barrier damage, protects mitochondrial function, and attenuates oxidative injury [30,31]. Our results showed that 3-MA effectively inhibited mitophagy and partially improved microglial phagocytic dysfunction and inflammation but failed to restore mitochondrial quality, representing a potential safety concern. In contrast, NBP effectively improved mitochondrial quality, stabilized membrane potential, and attenuated excessive mitophagy, yielding superior therapeutic effects. Regarding energy supply, NBP preserved more functional mitochondria and restored ATP production, supporting that microglial phagocytic dysfunction after cerebral ischemia arises from energy failure.

Due to the unique pathological microenvironment of cerebral ischemia, conventional drug delivery systems exhibit poor brain targeting. We therefore developed a microglia-derived exosomal system encapsulating NBP. Based on homotypic targeting, exosomes efficiently accumulate in ischemic lesions and target microglia, enabling targeted NBP delivery to mitigate excessive mitophagy. Tail vein injection of BV2exo@ICG-NBP systems confirmed effective accumulation in ischemic regions up to 24 h post-administration.

Previous studies often reported beneficial effects of enhanced mitophagy in cerebral ischemia [[22], [23], [24],^32^]. However, some studies showed that pretreatment with the mitophagy inhibitor 3-MA reduced infarct volume, whereas administration at the onset of reperfusion exacerbated injury [33], confirming the context-dependent complexity of mitophagy. We propose that these discrepancies arise because most studies used reperfusion models. Reperfusion restores oxygen and nutrient supply, sustaining basal energy metabolism such that mitophagy generally remains adaptive and protective. In contrast, our study demonstrates that during ischemia, excessively activated mitophagy induces cellular dysfunction via energy depletion. Microglial phagocytosis of apoptotic debris upregulates Drp1, further exacerbating mitophagy, which explains the prominent mitophagy signal observed in microglia at the single-cell level.

In summary, this study demonstrates that microglial phagocytosis during cerebral ischemia induces Drp1 upregulation, which triggers excessive mitophagy and subsequent energy failure, leading to phagocytic dysfunction and neuroinflammation. Inhibition of mitophagy alleviates these processes but fails to eliminate dysfunctional mitochondria, risking secondary damage. In contrast, NBP stabilizes mitochondrial membrane potential, ameliorates excessive mitophagy, and sustains energy supply, conferring superior neuroprotection.

## Author contribution

**Bangyue Wang:** Conceptualization, Methodology, Investigation, Data Curation, Writing – Original Draft, Visualization.

**Muchen Xi:** Methodology, Investigation, Formal Analysis, Data Curation, Writing – Original Draft.

**Keyi Lin:** Investigation, Resources, Data Curation, Validation.

**Qinglong Guo:** Conceptualization, Supervision, Funding Acquisition, Project Administration, Writing – Review & Editing, Resources.

**Yuyang Wang:** Conceptualization, Supervision, Funding Acquisition, Project Administration, Writing – Review & Editing, Corresponding Author.

**Note:** Bangyue Wang and Muchen Xi contributed equally to this work and share first authorship.

## Funding Statement

This research was supported by the Research Fund of Anhui Institute of Translational Medicine (Grant No. 2023zhyx-C68) and the project of Anhui Province Key Laboratory of Cognition and Neuropsychiatric Disorders, The First Affiliated Hospital of Anhui Medical University (Grant No. 2025KLCND01). The funding sources had no involvement in study design, data collection, analysis and interpretation, writing of the report, or the decision to submit the article for publication.

## Declaration of competing interest

The authors declare that they have no known competing financial interests or personal relationships that could have appeared to influence the work reported in this paper.
